# Recirculating Air Filtration Significantly Reduces Exposure to Airborne Nanoparticles

**DOI:** 10.1289/ehp.11169

**Published:** 2008-03-26

**Authors:** David Y.H. Pui, Chaolong Qi, Nick Stanley, Günter Oberdörster, Andrew Maynard

**Affiliations:** 1 Particle Technology Laboratory, University of Minnesota, Minneapolis, Minnesota, USA; 2 Environmental Medicine, University of Rochester, Rochester, New York, USA; 3 Woodrow Wilson Center for Scholars, Washington, DC, USA

**Keywords:** automobile, filtration, nanoparticle exposure, prevention, workplace

## Abstract

**Background:**

Airborne nanoparticles from vehicle emissions have been associated with adverse effects in people with pulmonary and cardiovascular disease, and toxicologic studies have shown that nanoparticles can be more hazardous than their larger-scale counterparts. Recirculating air filtration in automobiles and houses may provide a low-cost solution to reducing exposures in many cases, thus reducing possible health risks.

**Objectives:**

We investigated the effectiveness of recirculating air filtration on reducing exposure to incidental and intentionally produced airborne nanoparticles under two scenarios while driving in traffic, and while generating nanomaterials using gas-phase synthesis.

**Methods:**

We tested the recirculating air filtration in two commercial vehicles when driving in traffic, as well as in a nonventilation room with a nanoparticle generator, simulating a nanomaterial production facility. We also measured the time-resolved aerosol size distribution during the in-car recirculation to investigate how recirculating air filtration affects particles of different sizes. We developed a recirculation model to describe the aerosol concentration change during recirculation.

**Results:**

The use of inexpensive, low-efficiency filters in recirculation systems is shown to reduce nanoparticle concentrations to below levels found in a typical office within 3 min while driving through heavy traffic, and within 20 min in a simulated nanomaterial production facility.

**Conclusions:**

Development and application of this technology could lead to significant reductions in airborne nanoparticle exposure, reducing possible risks to health and providing solutions for generating nanomaterials safely.

Research over the past 15 years has demonstrated a close association between inhalation of airborne particles and increased pulmonary and cardiovascular disease (Mills et al. 2007; Pope and Dockery 2006; Seaton et al. 1995). Substantial attention has been given to airborne particulate matter < 2.5 μm (Dockery et al. 1993), yet there is increasing evidence that particles < 100 nm in diameter—referred to as ultrafine particles (UFPs) or nanoparticles—may play an important role in determining the health impact of inhaled aerosols (Oberdörster et al. 2007). Several studies have demonstrated that the potency of inhaled nanoparticles can be associated with size-related parameters, including surface area, rather than the more conventional exposure metric of mass concentration [reviewed by Oberdörster et al. (2005)]. Moreover, by virtue of their size, nanoparticles have the potential to move from the portal of entry (e.g., the respiratory tract) to secondary organs usually inaccessible to inhaled particles, including the brain (Elder et al. 2006; Oberdörster and Utell 2002; Semmler et al. 2004). They may also perturb key biologic processes, resulting in protein misfolding (Linse et al. 2007), a pathology involved in neurodegenerative disorders.

Some of the highest potential exposures to nanoparticles occur in cars, while driving and standing in heavy traffic. A recent study estimated that 33–45% of total UFP exposure for Los Angeles, California, residents occurs when traveling in vehicles (Fruin et al. 2008). Epidemiologic data show an association between exposure in traffic and the onset of a myocardial infarction within 1 hr afterward (Peters et al. 2004), and a recent study shows brief exposure to combustion-derived nanoparticles to promote myocardial ischemia in men with stable coronary disease (Mills et al. 2007). Emissions and exposure are dominated by nanoscale particles: Approximately 90% of emitted particles from diesel-powered cars on the road measure between 5 nm and 300 nm (Kittelson et al. 2004; Yao et al. 2006) (Figure 1), and emissions from spark-ignition engines show similar size distributions (Kittelson 1998). On the basis of number concentration, most of these particles are ≤20 nm (Figure 1). These particles have high deposition efficiency throughout the respiratory tract [see Supplemental Material, Figure 1 (http://www.ehponline.org/members/2008/11169/suppl.pdf)]. Based on mathematical model calculations and confirmed by experimental data, during nasal breathing > 50% of inhaled particles < 5 nm are deposited in the nasopharyngeal area of the human respiratory tract, approximately 35% of inhaled particles between 5 and 10 nm are depositing in the tracheobronchial area, and approximately 50% of inhaled particles of 20 nm are depositing in the gas-exchange (alveolar) region of the lung. The high deposition in the nasal region can potentially result in their translocation to the central nervous system via the olfactory nerve (Elder et al. 2006). Preventing such exposures can be of tremendous benefit, given that results of epidemiologic and toxicologic studies have associated traffic-related UFPs with adverse cardiovascular pulmonary effects (Penttinen et al. 2001; Peters et al. 2004), and toxicologic studies in animals have provided evidence for causality (Dick et al. 2003; Donaldson and Stone 2003).

Potential exposure to airborne nanoparticles is also being viewed as a significant issue in the burgeoning nanotechnology industry, particularly where nanometer-scale powders are produced using gas-phase synthesis (Maynard and Kuempel 2005). Although it remains unclear to what extent mechanisms of action and disease end points will converge between incidental and intentionally produced nanoparticles, effective methods of controlling exposures are likely to apply equally well to both categories of particles. For example, a recent intervention study using filtration of indoor air for only 48 hr reduced particle number concentrations from about 10^4^ particles/cm^3^ to about 3 × 10^3^ particles/cm^3^. This resulted in improved endothelial function in elderly people as measured by a significant increase in flow-mediated digital vasoresponse (Bräuner et al. 2008).

Car manufacturers are increasingly installing in-car (or cabin) air filters to reduce driver and passenger exposure to airborne particulates. Already, 100% of new cars in Europe and Japan and > 60% of new vehicles in the United States, China, and Korea have cabin air filters. However, the current International Standards Organization cabin air filter test standard does not require filtration efficiency to be evaluated for particles < 300 nm (International Standards Organization 2001). This test standard is primarily relevant for road dust and pollens and provides little to no information on how effective cabin air filters are for reducing exposure to nanoparticles from engine exhaust emissions (as shown in Figure 1). Zhu et al. (2007) studied the in-cabin exposure to UFPs in three different vehicles and concluded that car age and ventilation can significantly influence in-car exposure. They observed that setting ventilation to recirculation helped to reduce the exposure. However, beyond that there has been no systematic investigation of whether these filters provide significant protection.

In principle, even an inexpensive and relatively inefficient air filter may provide significant reductions in exposure if the air is continuously circulated through it. In addition to having implications to controlling nanoparticle exposure while driving, the same principle may be applicable to reducing exposures while generating engineered nanomaterials.

In this study, we evaluated the on-road effectiveness of in-cabin air recirculation systems in reducing driver and passenger nanoparticle exposure in two commercial vehicles. Using these data, we have developed an empirical model for nanoparticle concentration reduction using recirculation air filtration and evaluated the model against experimental data of nanoparticle concentration reductions while generating airborne silver nanoparticles in an enclosed area.

## Materials and Methods

The two commercial vehicles we used were a Saab 93 (2003 model) and a Toyota Camry (2007 model). On-road tests were carried out using both cars while driving in heavy traffic. When the ventilation system was set at medium fan speed with recirculation air off, a comparison of simultaneously measured aerosol concentration inside and outside of the cabin of the Saab 93 shows only a 46% particle removal efficiency [see Supplemental Material, Figure 2 (http://www.ehponline.org/members/2008/11169/suppl.pdf)]. With the ventilation system in recirculation mode, most of the cabin air recirculates, and a small (but unquantified) amount of outside air enters the cabin through natural infiltration. The Saab 93 is typical of many commercial vehicles in that recirculated air does not pass repeatedly through the cabin air filter. In contrast, air in the Toyota Camry passes through the filter each time it recirculates.

We used a portable condensation particle counter (CPC; model 3007, TSI Inc., Shoreview, MN) to measure particle number concentration inside the vehicle’s cabin, while it was driven through traffic. The CPC measures the number concentration of particles > 10 nm in diameter and smaller than approximately 1 μm. For all the tests, recirculation air was switched on when the in-cabin particle number concentration was about 50,000 particles/cm^3^, an average and typical concentration observed in this study when recirculation was off, and the subsequent variation of the particle number concentration was recorded by the CPC. All measurements were made while the vehicles were being driven on the road during heavy traffic. Measurements were made with and without the filter in place in the Toyota Camry, to assess the role of in-filter particle collection, compared with collection attributed to other mechanisms.

To investigate how recirculating air filtration affects particles of different sizes, we investigated the evolution of the particle size distribution in recalculating air for the Toyota Camry with the filter in place. We made time-resolved aerosol size distribution measurements using an engine exhaust particle sizer (model 3090; TSI Inc.). This instrument is able to rapidly measure particle size distributions in the nanometer range.

Extending the air recirculation filtration concept to workplaces where nanoparticles are intentionally generated, we measured reductions in aerosol concentration in a non-ventilated room (280 m^3^ in volume) where a silver nanoparticle aerosol generator (Scheibel and Porstendörfer 1983) was used to produce silver nanoparticles, simulating a gas-phase synthesis reactor leak. An open-loop wind tunnel was used to recirculate the room air through a Viledon HVAC filter (Freudenberg Nonwovens L.P., Hopkinsville, KY) at a rate of 50,970 L/min. We carried out tests for three scenarios to simulate different leaking conditions: *a*) The aerosol generator stops when turning on the recirculating air; *b*) the aerosol generator keeps generating and releasing particles at rates of 4.5 × 10^6^ particles/sec; and *c*) the aerosol generator keeps generating and releasing particles at rates of 1.71 × 10^7^ particles/sec. We continuously recorded particle number concentration by an ultrafine CPC (model 3025A; TSI Inc.).

## Results and Discussion

The filtration efficiencies of the cabin air filter measured in a laboratory wind tunnel test [see Supplemental Material, Figures 3 and 4 (http://www.ehponline.org/members/2008/11169/suppl.pdf)] as a function of particle size demonstrate a characteristic V-shaped curve (Hinds 1999), with a minimum filtration efficiency at approximately 350 nm. This particle size is commonly referred as the most penetrating particle size. Smaller particles (10–100 nm) are captured mainly by diffusion, and larger particles (1–10 μm), by interception and impaction mechanisms. The minimum efficiency was 22.9% at a flow rate equivalent to a medium ventilation system setting (filter face velocity of 10.8 cm/sec) and 17.4% at a flow rate equivalent to the highest ventilation system setting (filter face velocity of 21.5 cm/sec). This result indicates that the cabin air filter installed by the car manufacturer is relatively inefficient [compared with a high-efficiency particulate air (HEPA) filter].

Figure 2 shows in-cabin aerosol concentration with time for each vehicle (plotted concentration values before time = 0 were measured before recirculation was turned on). For comparison, the aerosol concentration measured using a CPC (model 3007; TSI Inc.) in a well-ventilated office (averaging 4,000 particles/cm^3^) is indicated on the plot. This is a typical concentration for such an environment measured in this study.

With air recirculation on and the filter in place, in-cabin aerosol concentration in the Toyota Camry was reduced to below typical office air concentrations in approximately 3 min. Without the filter installed, aerosol concentration still decreased exponentially, taking 13 min to reach 4,000 particles/cm^3^, suggesting significant collection within the ventilation and recirculation system. Results for the Saab 93 were between the two plots for the Toyota Camry, with aerosol concentration reaching 4,000 particles/cm^3^ in 9–10 min.

A recirculation model was developed to describe the variation of in-cabin particle concentration with time when using air recirculation:


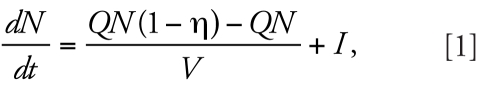


where *N* is the particle number concentration, *Q* is the ventilation flow rate, *V* is the cabin volume, η is the average one-pass particle removal efficiency, and *I* is the rate of particles entering the controlled volume from any source or infiltration—that is, from air bypassing the ventilation system entirely. This model assumes that the particle concentration is uniform in the test environments, and the variation of *I* is neglected. The particle removal efficiency in this model, η, is an average value for the sampling time.

Solving equation [1] enables particle concentration to be expressed as


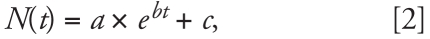


where *a* = *N*_0_ − *c*, *b* = −(*Q*/*V* )/η, and *c* = *I* × (*V*/*Q*η). *N*_0_ is the initial particle number concentration and is equal to the sum of parameters *a* and *c*. Fitting the experimental data by Equation 2 provides fitting parameters *a*, *b*, and *c*. With these fitting parameters, a known ventilation flow rate *Q*, and cabin volume *V*, the average particle removal efficiency η and the particle infiltration rate *I* could be calculated. As shown in Figure 2, the model can fit all the test data very well. For the tested Saab 93, *Q* was 60 L/sec and *V* was 2.55 m^3^, giving a recirculation time (*V*/*Q*) of 43 sec. For the tested Toyota Camry, *Q* was 73.6 L/sec and *V* was 2.86 m^3^, giving a recirculation time (*V*/*Q*) of 39 sec. Both cars have similar recirculation time (defined as *V*/*Q*) and thus should have similar concentration decay characteristics. Fitting the data gives particle removal efficiency η as 45.5% for the Camry and 27.2% for the Saab, reflecting the presence of the filter in the recirculation loop in the Camry, and its absence within the recirculation loop in the Saab.

Aerosol particles in both cars were clearly removed from the cabin air during recirculation by mechanisms other than filtration, which are most likely associated with intrinsic collection in the blower housing and cooler. We estimated these filter-independent losses within the Camry to give a particle removal efficiency of 19.1% from data without the filter installed. With the cabin air filter placed back in the recirculation loop in the Camry, the particle removal efficiency increased substantially to 45.5%, enabling aerosol concentrations to reach typical office levels approximately four times faster.

**Figure 1:**
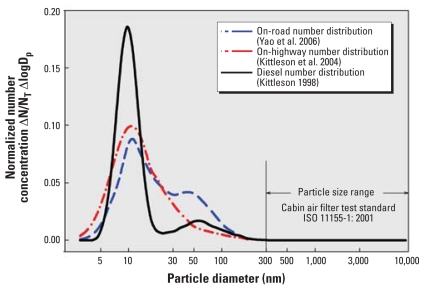
Measured diesel engine emission and on-road aerosol particle size distributions. ISO, data from International Standards Organization (2001). D_p_, particle diameter; N, normalized particle concentration in size bin ΔlogD_p_; N_T_, normalized particle total concentration.

**Figure 2:**
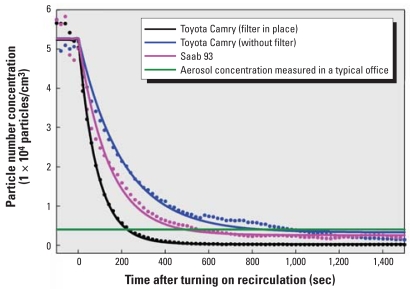
Measured in-cabin particle number concentration with time for a Toyota Camry and a Saab 93 while driving in heavy traffic, with the air ventilation system in recirculation mode. Dotted lines, experimental data. Solid lines, data fit using the developed empirical model. For comparison, the aerosol particle number concentration measured in a typical office is shown (4,000 particles/cm^3^).

**Figure 3:**
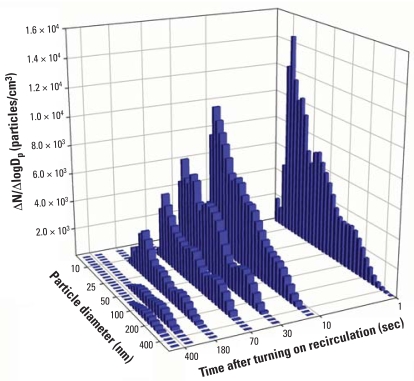
Measured variations in particle size distribution inside a Toyota Camry, with the air ventilation system in recirculation mode and the air filter in place. D_p_, particle diameter; N, particle number concentration in size bin ΔlogD_p_.

Figure 3 shows the time-resolved in-cabin aerosol size distribution during recirculation. Starting from an aerosol size distribution similar to those shown in Figure 1, particle concentrations of all the sizes were reduced continuously during the recirculation. Aerosol particles < 100 nm were rapidly removed from the air by the recirculation ventilation system. As would be expected from filtration theory, particles close to the filter’s most penetrating particle size were removed with the least efficiency, but still experienced significant reductions. This result demonstrates that the recirculating air filtration is especially effective in reducing exposure to UFPs even with a relatively inefficient air filter.

**Figure 4:**
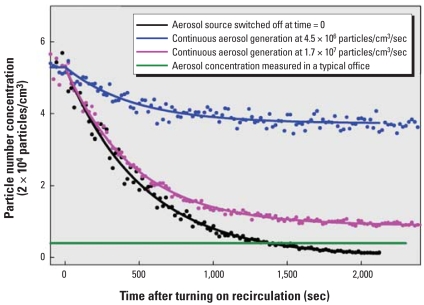
Measured particle number concentration in an enclosed room containing a source of silver nanoparticles with time, while recirculating air through an HVAC filter at 50,970 L/min. Dotted lines, experimental data. Solid lines, data fit using the developed empirical model. For comparison, the aerosol particle number concentration measured in a typical office is shown (4,000 particles/cm^3^).

Figure 4 shows the in-room aerosol concentration with time for the three simulated nanoparticle-leaking scenarios. The nanoparticles released from the aerosol generator have a lognormal size distribution with the geometric mean diameter of 18.2 nm and geometric standard deviation of 1.4. Once the nanoparticle generator was turned off, in 22 min the recirculation system reduced the aerosol concentration by a factor of 12.5, from about 50,000 particles/cm^3^ to 4,000 particles/cm^3^. With the generator running continuously at 4.5 × 10^6^ particles/sec, particle concentration dropped by a factor of 5.8 at 25 min after switching on the recirculation system; increasing the nanoparticle generation rate to 1.71 × 10^7^ particles/sec led to a reduction in concentration of only a factor of 1.4 after 25 min. In all cases, the previously developed model fit the data well. The average one-pass particle removal efficiency calculated from the curve fitting and model is 67.99% ± 4.94% for the three scenarios, demonstrating a very reproducible result. This efficiency is also reasonably close to the measured filtration efficiency of the Viledon HVAC filter using the same test particles, 59.63%. The 8% higher efficiency calculated by the model occurs probably because the effective recirculating volume is less than the room volume; that is, not all the 280 m^3^ in the room was involved in the recirculation. Because this model is able to accurately predict the variation of particle concentration in recirculating air, it will be very helpful in designing a recirculating air filtration system to control the nanoparticle exposure.

## Conclusion

Our results demonstrate that using air recirculation can substantially and rapidly reduce exposure to airborne nanoparticles within enclosed spaces. Such reduction was found to result in significant improvement of vascular function in elderly citizens (Bräuner et al. 2008). Although our data suggest an easy-to-implement solution to reducing nanoparticle exposure while driving in traffic, they also show that using relatively inexpensive filters in recirculating air systems may provide nanomaterial manufacturers and users with an effective means of controlling human exposure to airborne nanoparticles. Such systems would have to be properly designed and appropriately evaluated. In an industry where many small companies are struggling to develop effective ways of working safely with engineered nanomaterials, air recirculation may offer an exposure–control solution that is relatively inexpensive to deploy and simple to implement, thereby representing an important contribution to occupational and environmental health protection.
